# Association of *IL10* Polymorphisms and Leprosy: A Meta-Analysis

**DOI:** 10.1371/journal.pone.0136282

**Published:** 2015-09-04

**Authors:** Lucia Elena Alvarado-Arnez, Evaldo P. Amaral, Carolinne Sales-Marques, Sandra M. B. Durães, Cynthia C. Cardoso, Euzenir Nunes Sarno, Antonio G. Pacheco, Francisco C. F. Lana, Milton Ozório Moraes

**Affiliations:** 1 Laboratório de Hanseníase, Instituto Oswaldo Cruz, Fiocruz, Rio de Janeiro, Brazil; 2 Departamento de Enfermagem Materno-Infantil e Saúde Pública, Universidade Federal de Minas Gerais, Belo Horizonte, Brazil; 3 Departamento de Medicina Clínica, Serviço de Dermatologia da Universidade Federal Fluminense, Rio de Janeiro, Brazil; 4 Programa de Computação Científica, Fiocruz, Rio de Janeiro, Brazil; Swinburne University of Technology, AUSTRALIA

## Abstract

Leprosy is a chronic infectious disease that depends on the interplay of several factors. Single nucleotide polymorphisms (SNPs) in host immune related genes have been consistently suggested as participants in susceptibility towards disease. Interleukin-10 (IL-10) is a crucial immunomodulatory cytokine in mycobacterial pathogenesis and especially the -819C>T SNP (rs1800871) has been tested in several case-control studies indicating association with leprosy risk, although a recent consensus estimate is still missing. In this study, we evaluated the association of the -819C>T SNP and leprosy in two new Brazilian family-based populations. Then, we performed meta-analysis for this polymorphism summarizing published studies including these Brazilian family-based groups. Finally, we also retrieved published studies for other distal and proximal *IL10* polymorphisms: -3575 T>A (rs1800890), -2849 G>A (rs6703630), -2763 C>A (rs6693899), -1082 G>A (rs1800896) and -592 C>A (rs1800872). Results from meta-analysis supported a significant susceptibility association for the -819T allele, with pooled Odds Ratio of 1.22 (CI = 1.11–1.34) and *P*-value = 3x10^–5^ confirming previous data. This result remained unaltered after inclusion of the Brazilian family-based groups (OR = 1.2, CI = 1.10–1.31, *P-*value = 2x10^–5^). Also, meta-analysis confirmed association of -592 A allele and leprosy outcome (OR = 1.24, CI = 1.03–1.50, *P*-value = 0.02). In support of this, linkage disequilibrium analysis in 1000 genomes AFR, EUR, ASN and AMR populations pointed to r^2^ = 1.0 between the -592C>A and -819C>T SNPs. We found no evidence of association for the other *IL10* polymorphisms analyzed for leprosy outcome. Our results reinforce the role of the -819C>T as a tag SNP (rs1800871) and its association with leprosy susceptibility.

## Introduction

Interleukin-10 (IL-10) is mainly secreted by monocytes and lymphocytes and exhibits an important immunomodulatory activity regulating mainly antibody secretion or inflammation, which if sustained could provoke tissue injury during chronic diseases [1].

Leprosy, caused by intracellular pathogen *Mycobacterium leprae*, can only progress to active disease in a fraction of infected individuals. A sustained IL-10 production, although increases phagocytosis in macrophages can drive a permissive anti-microbial programming that leads to intracellular *M*. *leprae* replication [2]. In fact, CD163^+^ phagocytic phenotype is positively correlated with higher IL-10 levels in disseminated lepromatous patients [2, 3]. Among exposed household contacts with longer patient exposition a lower ratio of TNF/IL-10 was observed when compared to short term contacts [4].

Polymorphisms in the *IL10* promoter have been targeted of several case-control studies in an attempt to determine genetic markers that could associate to disease outcome [5]. The most common promoter polymorphisms include a distal group of three polymorphisms: -3575 T>A (rs1800890), -2849 G>A (rs6703630), -2763 C>A (rs6693899), and three proximal polymorphisms: -1082 G>A (rs1800896), -819 C>T (rs1800871) and -592 C>A (rs1800872) [6].

The first meta-analysis of *IL10* polymorphisms and association with leprosy was published in 2009 [7]. Several studies became available in the past few years testing other polymorphisms in the same gene as associated with leprosy. Our main purpose was to perform and updated meta-analysis on the -819 C>T polymorphism and include novel data based on transmission disequilibrium tests and to evaluate other *IL10* promoter polymorphisms using meta-analysis.

## Material and Methods

### Study subjects

We conducted two new association studies between the IL10 –819 C>T (rs1800871) polymorphism and leprosy susceptibility using family-based study designs. The first family-based study included a total of 443 individuals comprising 80 families recruited from Duque de Caxias, an hyperendemic municipality of Rio de Janeiro state (RJ) [8]. The second family based study enrolled 447 individuals in 119 families from Almenara municipality in Minas Gerais state (MG) [9]. Both states are located in the Southeastern region of Brazil. Data and sample collection protocols were accepted by local institutional review boards: Ethics Committee at Federal University in Rio de Janeiro state (HUCFF-UFRJ Protocol 187/04) and Ethics Committee at Federal University in Minas Gerais state (COEP-UFMG Protocol ETIC 454/10). All study participants provided written informed consent. Both familial samples included households of patients diagnosed with leprosy conformed mainly by trios formed by the index patient and their biological parents. Ethnicity of each subject was determined through morphological features and classified in either of three groups: Caucasoid, Mestizo and Black. General characteristics of both family-based studies are described in Table 1.

**Table 1:** Characteristics for the Brazilian family-based studies.

	Rio de Janeiro		Minas Gerais	
	Affected	Unaffected	Affected	Unaffected
Total individuals	198	245	176	271
Age (mean ± SD)	32 ±16	-	35.8 ± 13.3	54.8 ± 17.5
Sex				
Female	107 (0.54)	123 (0.50)	80 (0.45)	157 (0.58)
Male	91 (0.46)	122 (0.50)	96 (0.55)	114 (0.42)
Ethnicity^a^				
Caucasoids	81 (0.48)	75 (0.35)	26 (0.15)	54 (0.20)
Mestizoes	50 (0.30)	64 (0.30)	130 (0.74)	195 (0.72)
Blacks	37 (0.22)	75 (0.35)	19 (0.11)	22 (0.08)
Family-based structure^a^				
Total families	80		119	
1 affected sibling	54 (0.68)	-	98 (0.82)	-
2 affected siblings	11 (0.14)	-	16 (0.13)	-
>2 affected siblings	15 (0.18)	-	5 (0.04)	-
WHO classification^a^				
Paucibacillary	76 (0.61)	-	67 (0.48)	-
Multibacillary	48 (0.39)	-	72 (0.52)	-

Abbreviations: SD, standard deviation; WHO, World Health Organization.

^a^ Data is presented as total counts (frequency). The number of subject counts in ethnicity and WHO classification can differ from total individuals due to missing information.

### DNA extraction and SNP genotyping

We extracted DNA from frozen blood samples using a modified salting out procedure [10]. Genotyping for *IL10* –819 C>T (rs1800871) polymorphism was performed by real-time PCR using TaqMan probes (Life Technologies, EUA) C___1747362_10 and following manufacturer’s recommendations. We used 20–40 ng of DNA in each PCR reaction. Genotypes were determined by allelic discrimination in the StepOne real-time system software (Life Technologies, EUA).

### Literature search

We screened for published articles that evaluated the association between leprosy and *IL10* promoter polymorphisms in order to further perform meta-analysis. Literature search was made in databases such as MEDLINE using PubMed (http://www.ncbi.nlm.nih.gov/pubmed), Thomson Reuters Web of Science (http://wokinfo.com) and the Knowledge Resource Integrated Database from China—CNKI (http://www.cnki.net). Combinations of the keywords were used in the search as follows: “interleukin 10” and “leprosy”, “IL-10” and “leprosy”, “polymorphism(s)” and “leprosy”, “SNP(s)” and “leprosy”. Besides, when evaluating each article individually we also reviewed the reference lists and related citations suggested by Pubmed to broaden our results. We did not use specific SNP rs identification numbers to perform search. As inclusion criteria we considered studies if they were published up to February 2015 and provided genotypic data to calculate allelic counts in order to perform analysis. Studies were excluded if they were related to a previous publication or if control population deviated from Hardy-Weinberg equilibrium (HWE).

### Data extraction

Two authors (L.E.A.A. and E.P.A.) performed data extraction independently by following inclusion criteria as indicated above. The following information was recorded for each study: first author; year in which the study was published; the population that was evaluated; age and number of females and males for both cases and controls; the ratio between multibacillary and paucibacillary for case subjects, source of control individuals (household contacts or blood bank donors); genotyping method and, finally, genotype counts for cases and controls (S2 Table).

### Statistical analysis

Total genotypic counts for the -819 C>T polymorphism in the Brazilian family-based studies were characterized by direct counting. Association with leprosy was evaluated with the transmission disequilibrium test (TDT), which tests for deviations of the expected 50% frequency of transmission of the marker allele from heterozygous parents to the affected offspring. Whenever parental information was missing, we used sibling pairs to estimate genotypes. Analyses were performed as previously described using the software FBAT, version 2.0.2 [8, 11]. The proportion of the transmitted “risk” allele corresponding to the minor frequency allele (MA) was calculated with the tdthap package using R Software version 2.13.0.

### Meta-analysis

First, for case-control studies retrieved from literature, we used a Chi-square test to determine if genotype frequencies in controls groups of each study were distributed conforming to HWE [12]. Then, we used the methodology proposed by Kazeem et al., (2005) for performing combined meta-analysis of case–control and family-based studies in which is possible to obtain OR values from family-based studies estimated from the proportion of the transmitted high risk allele [13]. For case-control studies we determined total counts and frequency for both the “risk” allele and “non risk” allele. Publication bias was evaluated through Egger’s test in order to provide statistical evidence for funnel plot symmetry. Heterogeneity across studies was established by Cochran´s Q statistic. Pooled Odds Ratio (OR) estimates were obtained by DerSimonian and Laird random effects model in reference to the MA for each polymorphism. Concordantly, forest plots represent individual OR values for each study and pooled OR referring also to MA for the studied SNPs. In order to evaluate the influence of each study on the overall OR we performed sensitivity analyses in which one study is removed at a time. There was no adjustment for environmental effects or population stratification due to the lack of covariates to perform such analysis.

Specifically for rs1800871 (-819 C>T) we first conducted a meta-analysis considering only published studies and next we conducted a second analysis including our newly generated data from both Brazilian family-based studies. We considered *P-values* under 0.1 as significant for both assessing heterogeneity across studies and funnel plot asymmetry. Finally, association of *IL10* polymorphisms with leprosy susceptibility was significant with *P-values* <0.05. R Software version 2.13.0 with packages genetics, catmap and meta [14] were used for analyses.

### Linkage disequilibrium (LD) analysis across populations

We extracted data from the 1000 genomes browser (http://browser.1000genomes.org/index.html) encompassing the six *IL10* polymorphisms evaluated in this study. Individual genotypes corresponded to Phase 1 populations. Briefly, European population (EUR) is represented by 379 individuals from Europe with Western European Ancestry (Italy, England, Scotland, Finland and Spain), African population (AFR) totalizes 246 from Nigeria, Kenya and African Americans, Asian population (ASN) composed with 286 individuals from China and Japan and Amerindian group (AMR) is represented by 281 individuals from Colombia, Puerto Rico and Mexican Ancestry [15]. We used Haploview software to perform analysis and LD was evaluated trough r^2^ statistics [16] in each of the above mentioned populations.

## Results

### Family-based studies

The corresponding TDT results evaluating the -819 C>T polymorphism in the two novel Brazilian family-based groups are described in Table 2. The frequency of the -819 T allele was the same for both population (MA = 0.36). There was no significant differences between transmission of the risk T allele to affected offspring for the RIO (Z = -0.033, *P-*value = 0.94) and MG (Z = 0.114, *P-value* = 0.91) family-based populations, therefore we found no evidence of association between leprosy susceptibility and this polymorphism.

**Table 2:** Summary of the results from the family-based association studies with -819 C>T (rs1800871) and association with leprosy.

Population	Allele Frequency	Transmission Disequilibrium Test		
	C/T	Trans/Not trans^a^	*Z*	*P-value*
**Rio de Janeiro**	0.64/0.36	29/32	-0.033	0.97
**Minas Gerais**	0.64/0.36	48/47	0.114	0.91

Abbreviations: Trans, Transmitted.

^a^ Trans = Transmission in reference to Minor or risk allele —819 T.

### Eligible studies for meta-analysis

**Fig 1:**
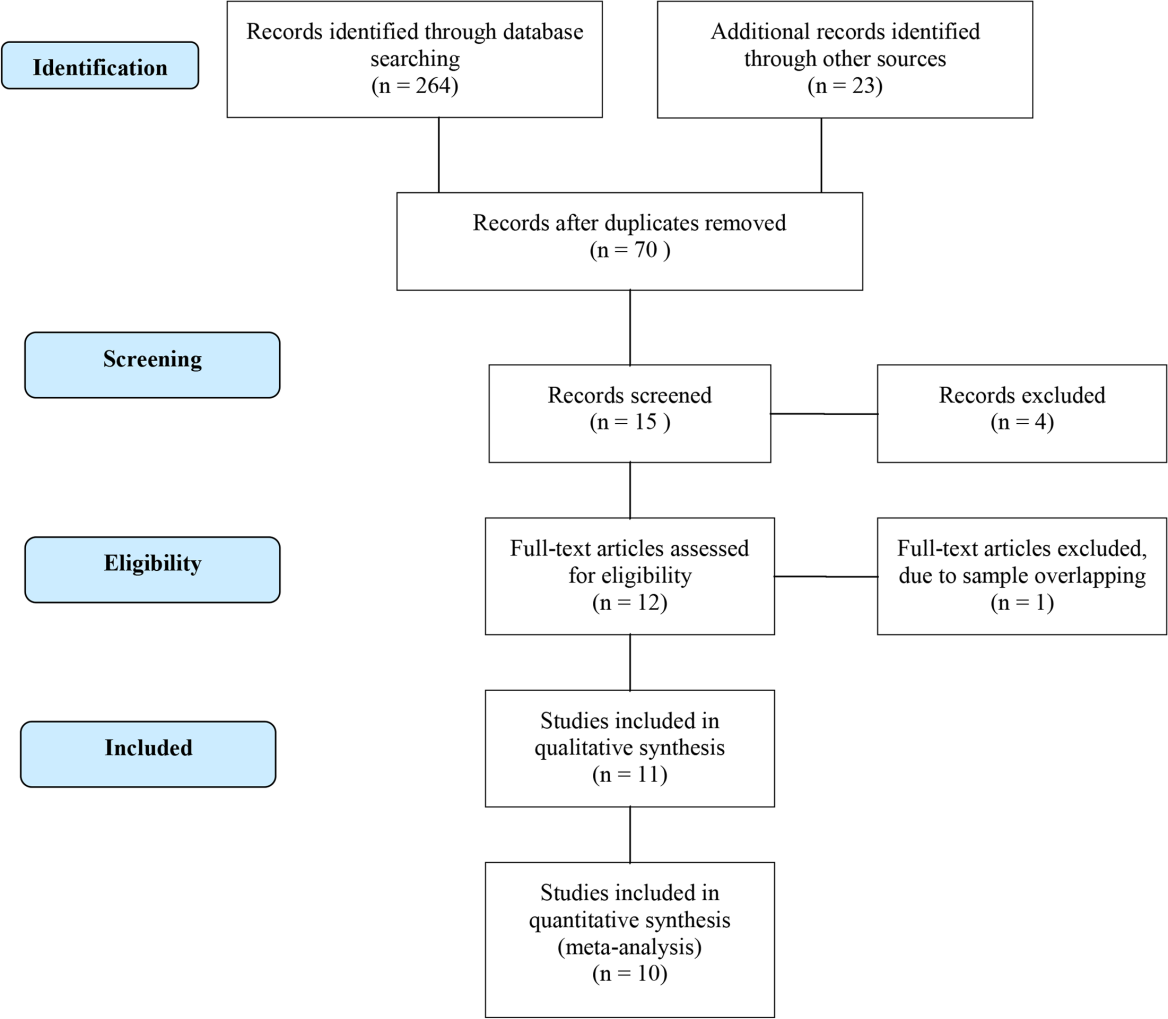
Flow diagram of the process of identification of eligible studies.

The flow diagram that allowed for identification of eligible studies is illustrated in Fig 1 [17].

We found a total of 11 studies that evaluated the influence of *IL10* polymorphisms and leprosy published between 2001–2015; all of them were case-control studies that were conducted in Brazilian [7, 18–20], Indian [21–23], Malawian [24], Mexican [25], Colombian [26] and Chinese [27] populations. The studies from Malhotra et al., 2005 and Aggarwal et al., 2011 presented partially overlapping population, therefore we excluded from meta-analysis the data from Malhotra et al 2005 [21] corresponding to the -819 and -592 polymorphisms. The included studies summarized a total of 2,941 cases and 3,763 controls. The general information regarding included studies is detailed in S2 Table. All control groups followed Hardy-Weinberg distribution, except for the -1082 polymorphism in which the studies [19], [7] and [26] were subsequently excluded from meta-analysis (Table 3).

**Table 3:** Summary of extracted data from the papers selected for the meta-analysis.

First author, year	Cases		Controls		
	Allele 1*	Allele 2	Allele 1	Allele 2	*P* for HWE
**-3575 T>A (RS1800890)**					
Moraes et al., 2004	154 (29)	374 (71)	122 (31)	270 (69)	0.51
Malhotra et al., 2005	140 (25)	424 (75)	130 (24)	402 (76)	1.0
Pereira et al., 2009	200 (27)	538 (73)	206 (27)	554 (73)	0.054
Chen et al., 2013	29 (8)	357 (92)	20 (5)	356 (95)	0.08
**-2849 G>A (RS6703630)**					
Moraes et al., 2004	129 (22)	465 (78)	106 (19)	460 (81)	0.24
Malhotra et al., 2005	55 (10)	509 (90)	66 (12)	466 (88)	0.15
Pereira et al., 2009	136 (19)	598 (81)	128 (17)	614 (83)	0.58
Chen et al., 2013	6 (2)	380 (98)	2 (1)	374 (99)	1.0
**-2763 C>A (RS6693899)**					
Moraes et al., 2004	182 (31)	398 (69)	117 (30)	267 (70)	0.50
Malhotra et al., 2005	140 (25)	424 (75)	130 (24)	402 (76)	1.0
Pereira et al., 2009	201 (27)	531 (73)	187 (25)	549 (75)	0.89
Chen et al., 2013	23 (6)	363 (94)	8 (2)	368 (98)	0.07
**-1082 G>A (RS1800896)**					
Moraes et al., 2004	183 (31)	405 (69)	182 (31)	400 (69)	0.002
Fitness et al., 2004	126 (33)	256 (67)	246 (35)	452 (65)	0.56
Malhotra et al., 2005	154 (27)	410 (73)	141 (27)	391 (73)	1.0
Pereira et al., 2009	215 (33)	427 (67)	226 (30)	518 (70)	<0.001
Velarde et al., 2012	187 (19)	813 (81)	173 (17)	847 (83)	0.16
Cardona et al., 2012	45 (23)	155 (77)	55 (28)	145 (72)	0.002
Garcia et al., 2013	103 (37)	173 (63)	60 (31)	132 (69)	1.0
Chen et al., 2013	44 (11)	342 (89)	27 (7)	349 (93)	0.24
Tarique et al., 2015	78 (38)	126 (62)	60 (25)	180 (75)	0.62
**-819 C>T (RS1800871)**					
Santos et al., 2002	153 (38)	251 (62)	39 (31)	85 (69)	0.77
Moraes et al., 2004	231 (39)	361 (61)	197 (34)	389 (66)	0.9
Fitness et al., 2004	152 (35)	278 (65)	242 (34)	464 (66)	0.24
Pereira et al., 2009	286 (39)	440 (61)	251 (34)	495 (66)	0.91
Aggarwal et al., 2011	760 (47)	854 (53)	1396 (43)	1884 (57)	0.96
Velarde et al., 2012	56 (44)	72 (56)	112 (41)	164 (59)	0.38
Cardona et al., 2012	64 (32)	136 (68)	85 (42)	115 (57)	1.0
Garcia et al., 2013	97 (35)	179 (65)	60 (31)	132 (69)	0.24
Chen et al., 2013	267 (69)	119 (31)	257 (68)	119 (32)	0.74
Tarique et al., 2015	141 (69)	63 (31)	126 (52)	114 (48)	1.0
**-592 C>A (RS1800872)**				
Santos et al., 2002	136 (34)	268 (66)	37 (30)	87 (70)	1.0
Fitness et al., 2004	149 (35)	275 (65)	241 (33)	483 (67)	0.41
Aggarwal et al., 2011	760 (47)	854 (53)	1393 (42)	1887 (58)	1.0
Cardona et al., 2012	64 (32)	136 (68)	85 (42)	115 (57)	1.0
Garcia et al., 2013	135 (49)	141 (51)	60 (31)	132 (69)	0.24
Chen et al., 2013	267 (69)	119 (31)	257 (68)	119 (32)	0.74

Results indicate allele counts (frequency) for each polymorphism. RA = Risk allele.

* Refers to risk allele for case-control studies.

Data for *IL10* –1082 G>A (rs1800896) polymorphism from Moraes et al., 2004, Pereira et al., 2009, Cardona et al., 2012 were excluded from analysis because control groups did not follow HWE.

### Meta-analysis

When testing for publication bias results did not indicate funnel plot asymmetry for none of the six *IL10* polymorphisms (*P-values* for Egger´s test > 0.1). Cochran´s Q statistic (Table 4) suggested heterogeneity across studies evaluating leprosy association with two of the polymorphisms: **-**1082 and -592 (*P-values* of 0.04 and 0.05 respectively). After removing one study at a time to assess its individual influence over the pooled result, we found no evidence of alteration in OR values for neither -3575 T>A (rs1800890), -2849 G>A (rs6703630), -2763 C>A (rs6693899), -1082 G>A (rs1800896) polymorphisms (data not shown).

**Table 4:** Meta-analysis results from studies investigating leprosy association and *IL10* promoter polymorphisms.

SNP	Risk Allele	N^a^	Meta-analysis			Heterogeneity	
			OR	95% CI	*P-value*	*Q*	*P-value*
**-3575 rs1800890**	A	4	0.99	0.86–1.15	0.95	1.92	0.59
**-2849 rs6703630**	A	4	1.05	0.81–1.36	0.69	5.25	0.15
**- 2763 rs6693899**	A	4	1.13	0.91–1.42	0.27	5.90	0.11
**-1082 rs1800896**	G	6	1.20	0.99–1.47	0.07	11.45	0.04
**-819 rs1800871**	**T**	**9 #**	**1.22**	**1.11–1.34**	**3x10** ^**–5**^	9.55	0.30
		11 *	**1.20**	**1.10–1.31**	**2x10** ^**–5**^	11.04	0.35
**-592 rs1800872**	**A**	**5 #**	**1.24**	**1.03–1.50**	**0.02**	9.60	0.05

Results correspond to random effects analysis.

Abbreviations: CI, Confidence Interval.

^**a**^N = Number of studies included in the meta-analysis for each polymorphism.

# Combined results only with literature studies excluding Cardona et al., 2012.

* Combined results including the two Brazilian family-based association studies

**Fig 2:**
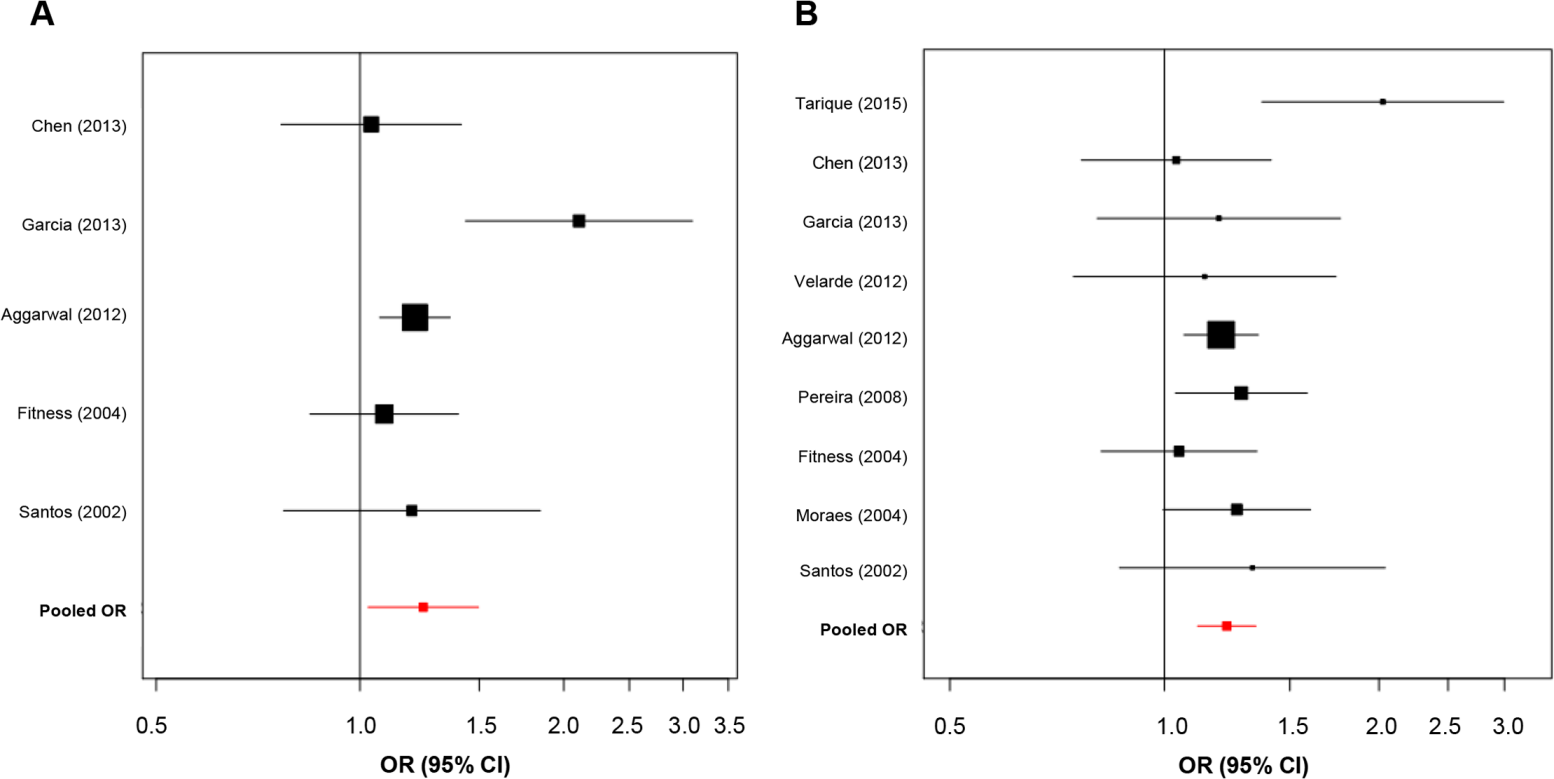
Forest plots summarizing association of *IL10* promoter polymorphisms and leprosy. **(A)** Forest plot for -592 C>A (rs1800872). Five case-control studies were evaluated under random-effects model. Bars represent 95% confidence interval and boxes represent OR values. **(B)** Forest plot for -819 C>T (rs1800871). Nine case-control studies were evaluated under random-effects model. Bars represent 95% confidence interval and boxes represent OR values.

Meta-analysis results (Table 4) showed a significant association of the -819 C>T (rs1800871) and -592 (rs1800872) polymorphisms with leprosy susceptibility. Amongst the published literature, Cardona et al., 2012 [26] presented OR results that diverged from pooled risk association (S1 Fig). The removal of study [26] during sensitivity analysis for the -819 polymorphism slightly increased the pooled OR from 1.18 (CI = 1.04–1.34) to 1.22 (CI = 1.10–1.31) and also improved the significance of *P-*value from 0.01 to 3x10^–5^. In the case of the -592 polymorphism, after exclusion of Cardona et al., 2012, *P-*value was altered to 0.02. Pooled OR values for the -592 polymorphism increased from 1.14 (CI = 0.91–1.43) to 1.24 (CI = 1.03–1.50) after removal of this study from meta-analysis. Therefore, after sensitivity analysis we considered this as an outlier study and decided to remove it from the final quantitative synthesis for -592C>A and -819C>T SNPs detailed in Table 4 and also visualized in Fig 2.

**Fig 3:**
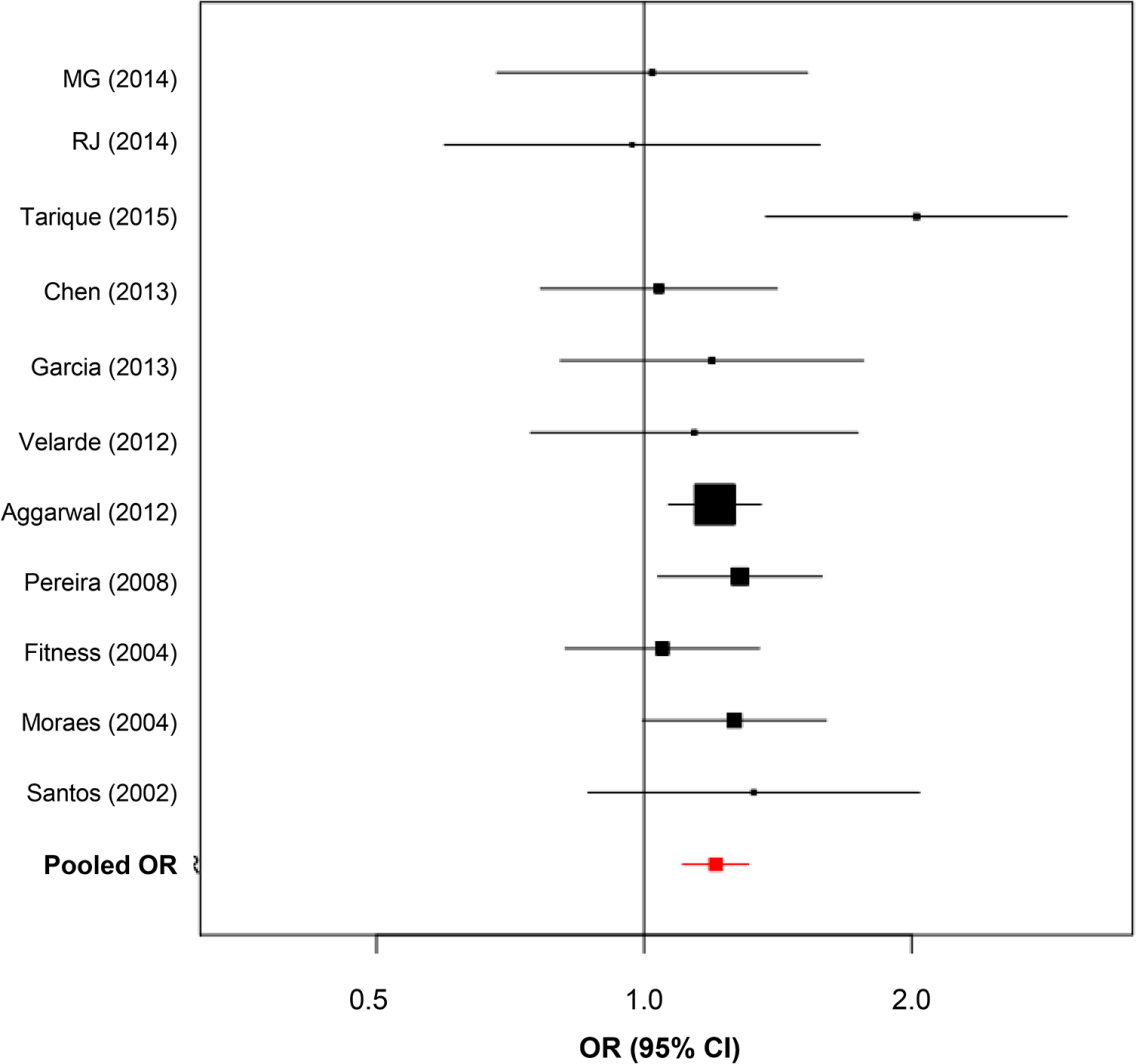
Forest plot for -819 C>T (rs1800871) including Brazilian Family-based data (MG and RJ). Bars represent 95% confidence interval and boxes represent OR values.

We found significant evidence of association with leprosy susceptibility and the -819 C>T (rs1800871) polymorphism first when considering the available articles from literature (Pooled OR = 1.22; CI = 1.10–1.31; *P-value =* 3x10^–5^). After inclusion of the two Brazilian family-based studies data for this SNP summarized 10 studies and pooled results remained similar with OR = 1.20, (95% CI = 1.10–1.31) and *P-*value = 2x10^–5^ reinforcing the role of the -819 T allele and leprosy susceptibility (Fig 3).

In contrast the remaining *IL10* polymorphisms: -3575 T>A (rs1800890), -2849 G>A (rs6703630), -2763 C>A (rs6693899) and -1082 G>A (rs1800896) showed no significant association with leprosy outcome (*P-*values >0.05) (S3–S6 Figs).

### Linkage disequilibrium (LD) analysis across populations

Finally to better understand these results, we performed linkage disequilibrium analysis to test for the presence of bins in the region encompassing these SNPs. LD plots for AFR, EUR, ASN and AMR populations from 1000 genomes (S7 Fig) presented perfect LD values (r^2^ = 1) between -819 (rs1800871) and -592 (rs1800872) polymorphisms. Specifically in ASN we also observed high LD between the -1082 (rs1800896) and -2763 (rs6693899) polymorphisms. For both EUR and AMR groups the distal polymorphism -3575 (rs1800890) had moderate LD values (r^2^>0.6) with —2763 (rs6693899) and -1082 (rs1800896) SNPs.

## Discussion

Our goal was to update the previous meta-analysis [7] which suggested the -819T allele as a marker of leprosy susceptibility. We included two previously unpublished Brazilian family samples and reviewed the literature retrieving novel studies. In total, we evaluated ten studies as compared to five in the previous study. When evaluating the results of the two new Brazilian family-based association studies we found no association with -819 T risk allele and leprosy outcome. However, when meta-analysis was performed combining literature data, results indicate a significant risk association for the -819 T allele with pooled OR of 1.18. The exclusion of an outlier study raised the pooled estimates to OR = 1.22. In the same reasoning, the -592 A allele showed a significant risk association with pooled OR of 1.24 after exclusion of study [26]. Our criteria for exclusion was corroborated with LD data that suggested clearly that -592C>A and -819C>T SNPs are linked in a group of reference populations from 1000 genomes database. Therefore, our meta-analysis results seemed to be in accordance and in the same direction for both variants. Although the association in this study suggested a modest risk of 20% for both -819T and -592A alleles in leprosy susceptibility, these results support the role of these polymorphisms on leprosy susceptibility replicating association amongst several populations with different ethnic background included in meta-analysis [7, 19, 21, 22, 24, 27] reinforcing the role of this cytokine in leprosy outcome. We did not find evidence of association for any other promoter polymorphisms when they were evaluated individually.

Interestingly, unrelated household contacts (HC) constituted the control group in the Cardona study, differently from control groups of other reviewed articles that consisted of blood bank (BB) donors or healthy individuals (HI). We cannot directly infer that the control group composition could influence the results, but some cryptic consanguineous relations between controls and cases could introduce bias.

Recently, two meta-analysis also showed that *IL10* –819C>T and -592C>A were associated with tuberculosis. Both studies pointed to the -819T allele as significantly associated in Asians when subgroup analyses were performed [28, 29]. The -592 polymorphism was associated with TB risk in Asians [28] differently from the second study that suggested association in European subgroup [29]. Unfortunately, as a limitation in this study, the number of combined studies for the *IL10* polymorphisms did not allow us to explore possible sources of heterogeneity such as subgroup analysis, this, for instance, could allow stratifying multibacillary or paucibacilary groups. It would be interesting that the publication of future studies evaluating such polymorphisms in well-defined clinical groups could aid in confirming results for distal polymorphisms and to perform clinical stratification analysis in proximal *IL10* polymorphisms.

## Conclusions

In the present study, we provided an updated pooled OR estimate strengthening previous findings for *IL10* promoter polymorphisms and its association with leprosy. A better understanding of the genomic arrangements of this region indicated perfect LD between -819C>T and -592C>A, and, consequently, both SNPs were associated with leprosy.

## Supporting Information

S1 Meta-Analysis Checklist Meta-analysis on genetic association studies form (PDF)

S1 Fig Forest plots summarizing association of *IL10* promoter polymorphisms and leprosy including ref [26] in analysis. **(A)** Forest plot for *IL10* –592 C>A (rs1800872). Bars represent 95% confidence intervals and boxes represent OR values. (**B)** Forest plot for *IL10* –819 C>T (rs1800871). Bars represent 95% and boxes represent OR values
(TIF)

S2 Fig Funnel plot for publication bias. **(A)** *IL10* –592 C>A (rs1800872). **(B)** *IL10* –819 C>T (rs1800871)
(TIF)

S3 Fig *IL10* –3575 T>A (RS1800890) and leprosy association. **(A)** Forest plot summarizing association. Bars represent 95% confidence intervals and boxes represent OR values. **(B)** Funnel plot for publication bias
(TIF)

S4 Fig *IL10* –2849 G>A (rs6703630) and leprosy association. **(A)** Forest plot summarizing association. Bars represent 95% confidence intervals and boxes represent OR values. **(B)** Funnel plot for publication bias
(TIF)

S5 Fig *IL10* –2763 C>A (rs6693899) and leprosy association. **(A)** Forest plot summarizing association. Bars represent 95% confidence intervals and boxes represent OR values. **(B)** Funnel plot for publication bias
(TIF)

S6 Fig *IL10* –1082 G>A (rs1800896) and leprosy association. **(A)** Forest plot summarizing association. Bars represent 95% confidence intervals and boxes represent OR values. **(B)** Funnel plot for publication bias
(TIF)

S7 Fig Linkage disequilibrium analysis for *IL10* polymorphisms in populations from 1000 genomes Phase 1. **(A)** African-AFR. **(B)** European-EUR. **(C)** Asian-ASN and **(D)** Amerindian-AMR. Values shown in each box and the intensity of shading are proportional to r^2^. *IL10* –592 C>A (rs1800872) and *IL10* –819 C>T (rs1800871) promoter polymorphisms are shown with *.
(TIF)

S1 Table PRISMA Checklist for Meta-analysis on genetic association studies. (PDF)

S2 Table Characteristics of the studies included in the meta-analysis for the *IL10* polymorphisms and leprosy. (DOC)
