# Supplementary material for: Association of IL10 Polymorphisms and Leprosy: A Meta-Analysis
Source: PLoS One. 2015 Sep 4;10(9):e0136282. doi: 10.1371/journal.pone.0136282 (PMC4560376; doi:10.1371/journal.pone.0136282)
Supplement: S1 Meta-Analysis Checklist — (PDF) [file pone.0136282.s001.pdf]

Meta-analysis on Genetic Association Studies Checklist | PLOS ONE

	Item	Section name and paragraph number within manuscript
	**Introduction**	
1	Provide a detailed justification for the polymorphism studied; if a single polymorphism was analyzed, give details as to why others were not included in the meta-analysis.	Paragraph 3.<br>Lines 51-55
2	Provide a detailed justification for the population(s) and clinical condition studied.	Paragraph 1 and 2.<br>Lines 56-60
	**Methods**	
3	Provide full details of the search strategy employed; outline the full electronic search strategy –specific combination of keywords and any limits applied- for at least one database. Specify whether synonyms of polymorphisms/genes (e.g. SNP number) were searched.	Literature search paragraph<br>Lines 88-96
4	Report full details on the inclusion and exclusion criteria applied for selecting studies. *Please list the excluded articles and the reasons for exclusion of each article in a supplementary file.*	Inclusion criteria indicated in literature search paragraph<br>Lines 96-98<br>Excluded studies indicated in Table 3
5	Provide details on how the quality of the studies included in the analyses was assessed.	Literature search paragraph<br>Line 99,100
6	Describe steps taken to contact study authors to identify additional studies and to request missing data.	Literature search paragraph<br>Lines 94,96
7	Describe how environmental effects were adjusted for, if this adjustment was not conducted, outline the reasons for this.	Meta-analysis paragraph<br>Lines 132,133
8	Describe the methods of handling heterogeneity/between-study variance.	Meta-analysis paragraph<br>Lines 126, 127
9	Describe how the Hardy-Weinberg equilibrium and linkage	Meta-analysis

	disequilibrium were assessed.	paragraph<br>Lines 120,121<br>LD assesment<br>Linkage disequilibrium paragraph<br>Lines 141-150
10	Describe and justify the choice of model for the analyses (per-allele vs per-genotype vs genetic model-free, random effects vs fixed effects).	Meta-analysis paragraph<br>Lines 120-124.<br>Lines 127-129
11	Describe whether a sensitivity analysis has been completed.	Meta-analysis paragraph<br>Lines 131
12	Describe whether an assessment of the effects of population stratification has been conducted.	Meta-analysis paragraph<br>Lines 132,133
13	Describe whether study-specific results have been assessed and if so the reasons for this (e.g. forest plot).	Meta-analysis paragraph<br>Lines 129
	**Results**	
14	Include flow diagram for the studies included in the meta-analysis as the first figure for the manuscript	Results<br>Line 164
15	Report details on allele/genotype prevalence.	Results<br>Table 3. Line 415
16	Report the effect size estimates and p values for each analysis.	Results<br>Table 4. Line 420
	**Discussion**	
17	Discuss the limitations of the meta-analysis, including genotyping errors/bias and publication bias.	Discussion<br>Lines 254-258
18	If the meta-analysis identifies an association within a subgroup of the population studied but not another, discuss the implications of these results, and if applicable the possibility of subgroup-specific publication bias.	Discussion<br>Lines 251-258
19	Discuss the suitability of the sample size employed to the	Discussion

	research question and the power of the study.	Lines 258-260
