# Supplementary figures and images for: Association of IL10 Polymorphisms and Leprosy: A Meta-Analysis
Source: PLoS One. 2015 Sep 4;10(9):e0136282. doi: 10.1371/journal.pone.0136282 (PMC4560376; doi:10.1371/journal.pone.0136282)

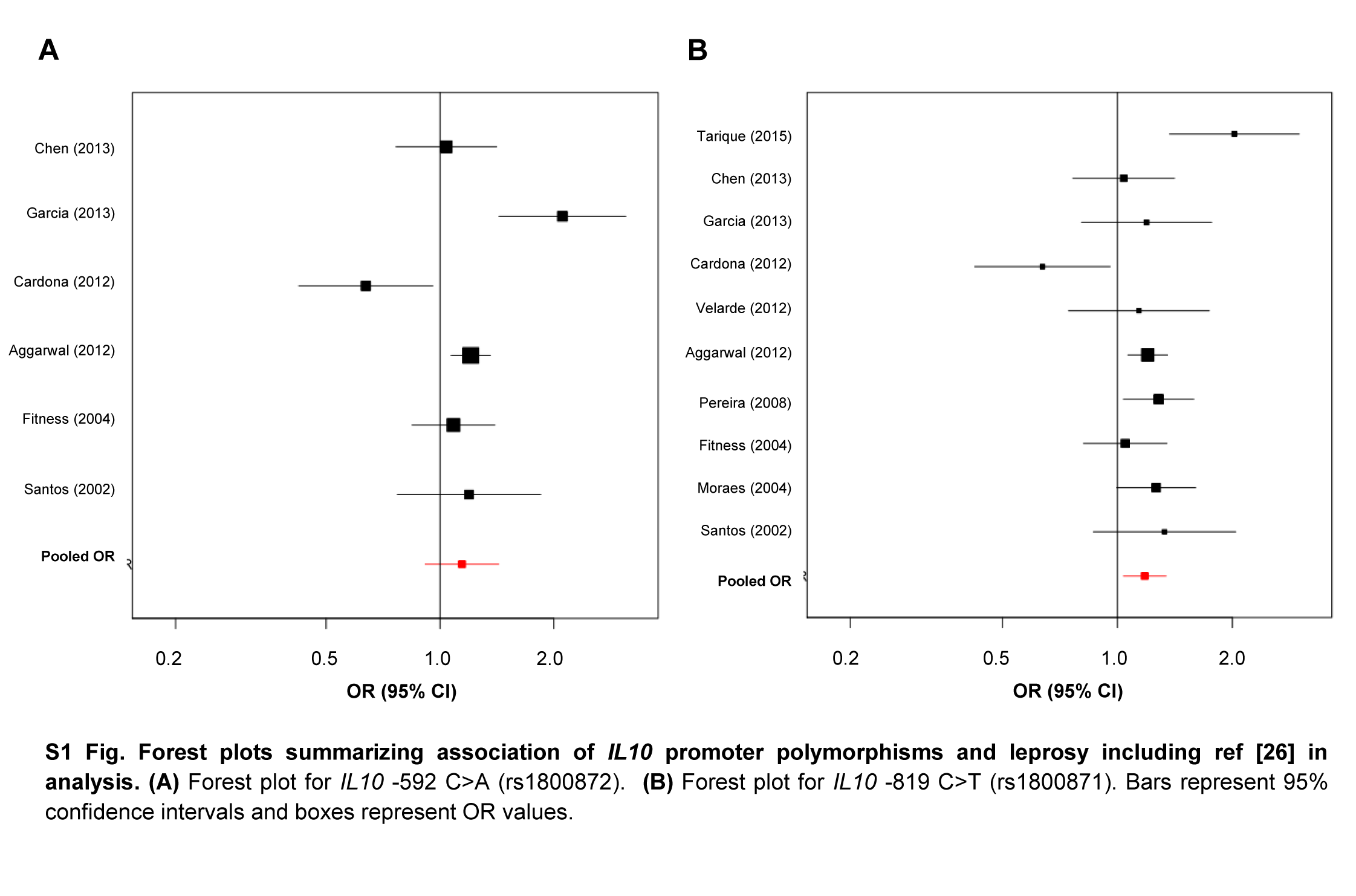

Supplement: S1 Fig — (A) Forest plot for IL10 –592 C>A (rs1800872). Bars represent 95% confidence intervals and boxes represent OR values. (B) Forest plot for IL10 –819 C>T (rs1800871). Bars represent 95% and boxes represent OR values (TIF) [file pone.0136282.s002.tif]

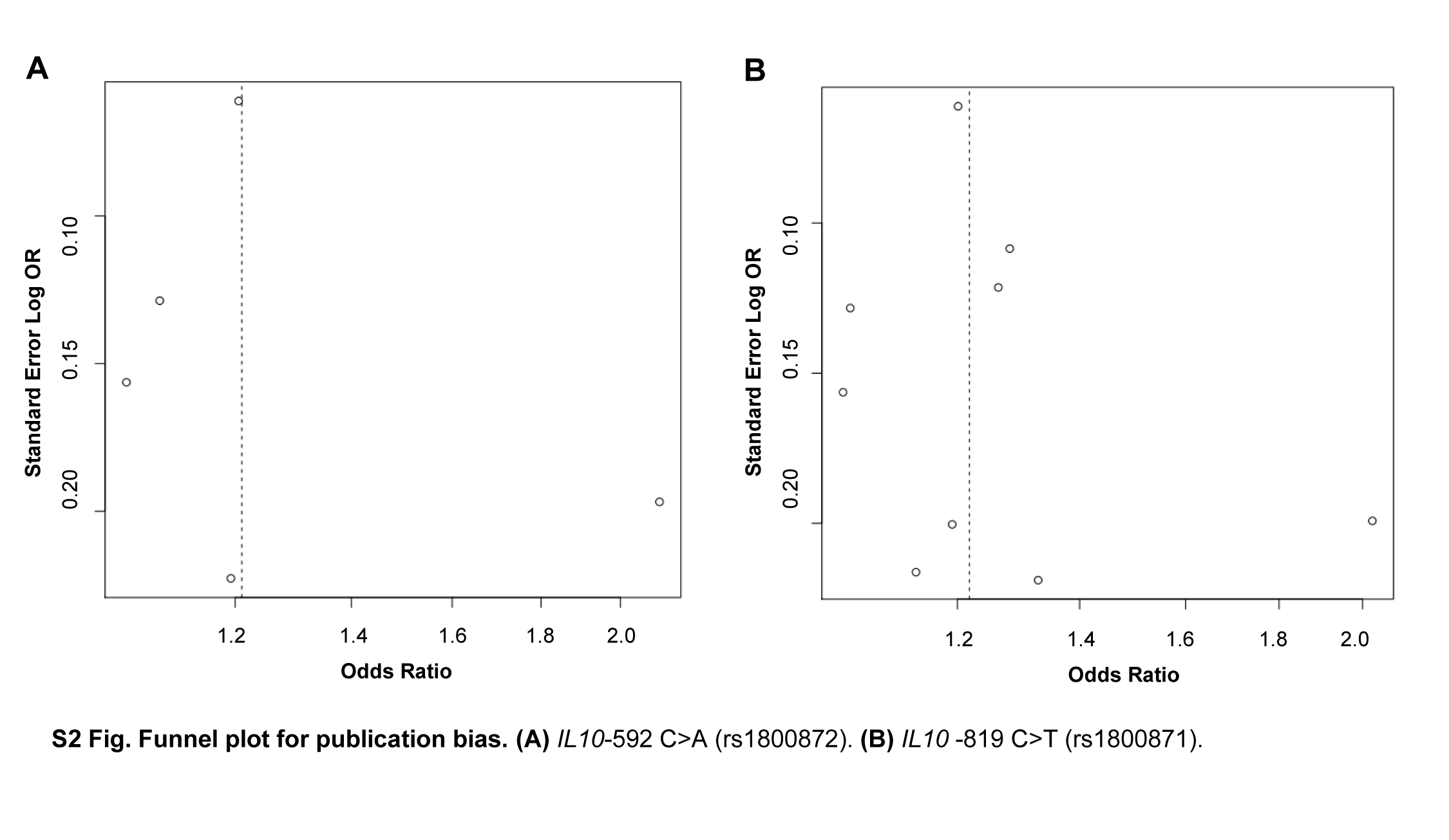

Supplement: S2 Fig — (A) IL10 –592 C>A (rs1800872). (B) IL10 –819 C>T (rs1800871) (TIF) [file pone.0136282.s003.tif]

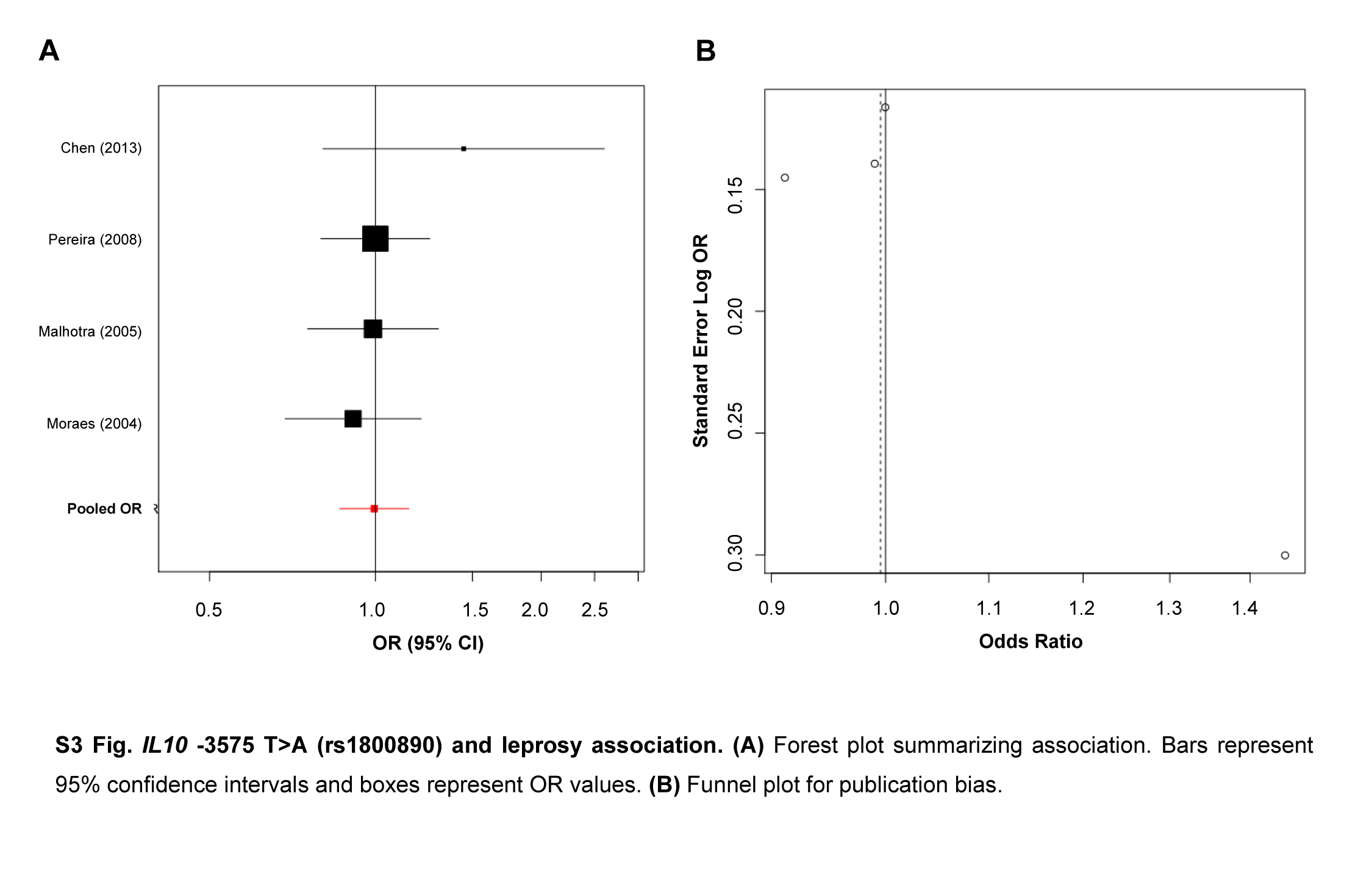

Supplement: S3 Fig — (A) Forest plot summarizing association. Bars represent 95% confidence intervals and boxes represent OR values. (B) Funnel plot for publication bias (TIF) [file pone.0136282.s004.tif]

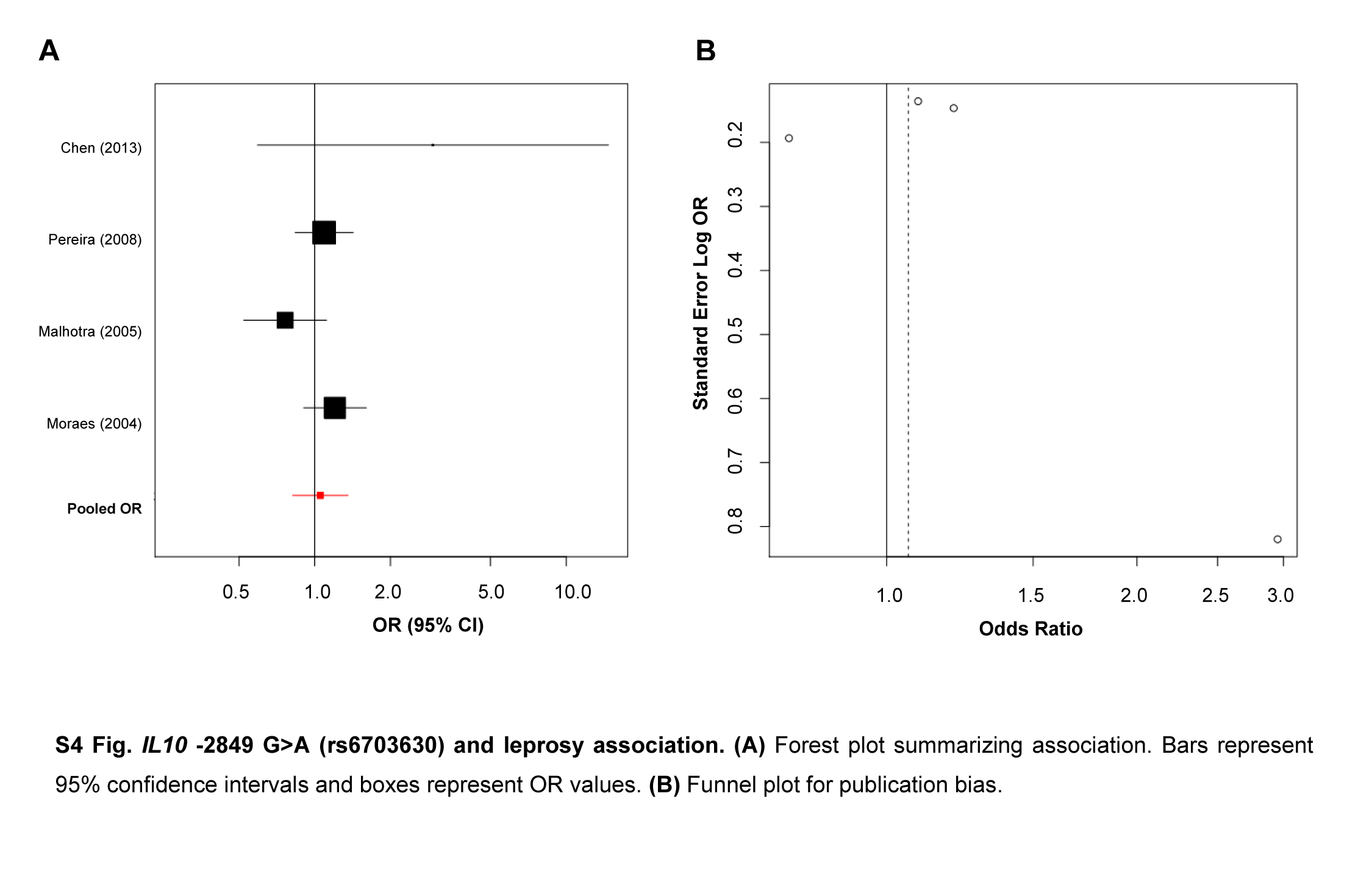

Supplement: S4 Fig — (A) Forest plot summarizing association. Bars represent 95% confidence intervals and boxes represent OR values. (B) Funnel plot for publication bias (TIF) [file pone.0136282.s005.tif]

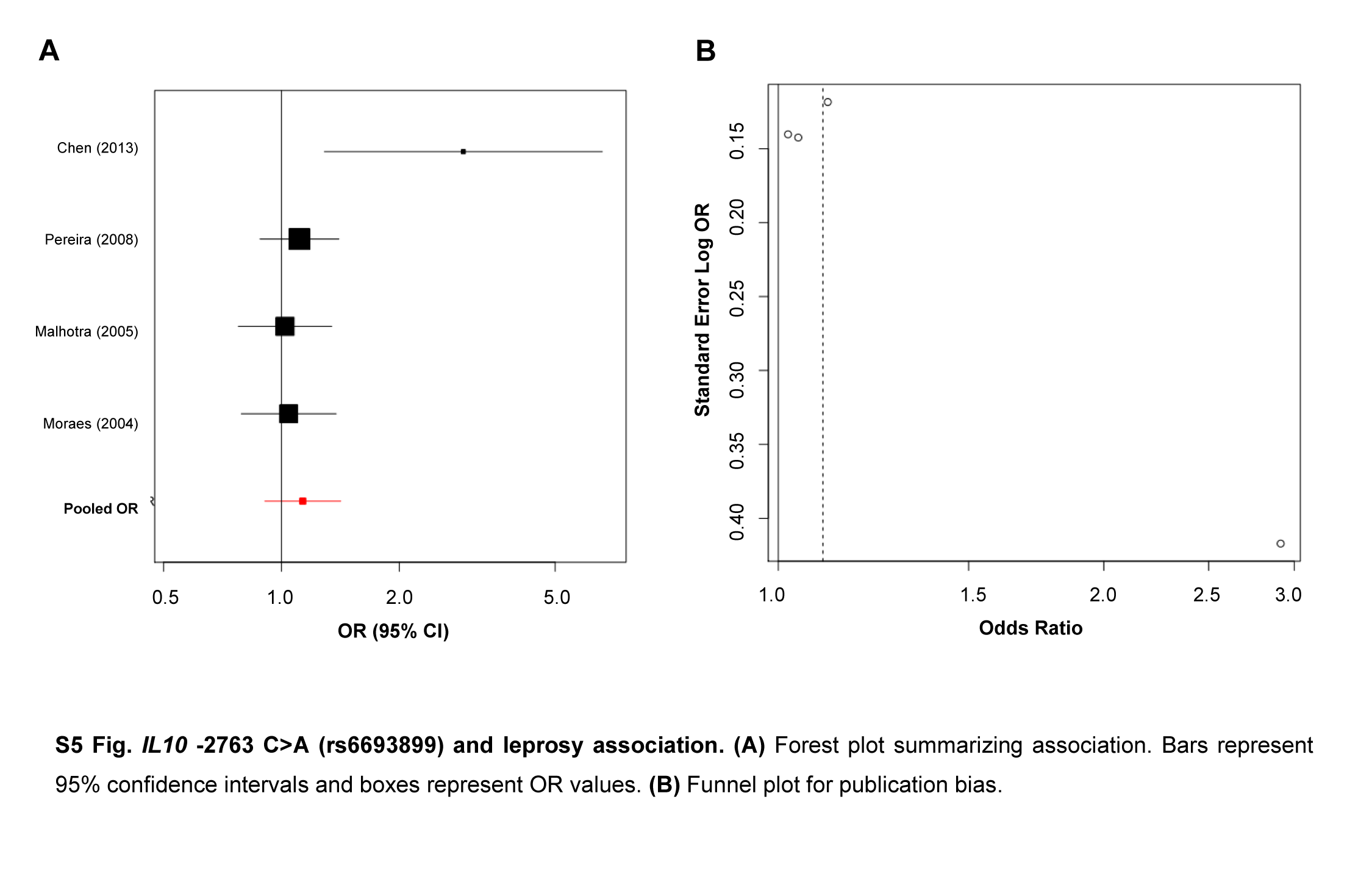

Supplement: S5 Fig — (A) Forest plot summarizing association. Bars represent 95% confidence intervals and boxes represent OR values. (B) Funnel plot for publication bias (TIF) [file pone.0136282.s006.tif]

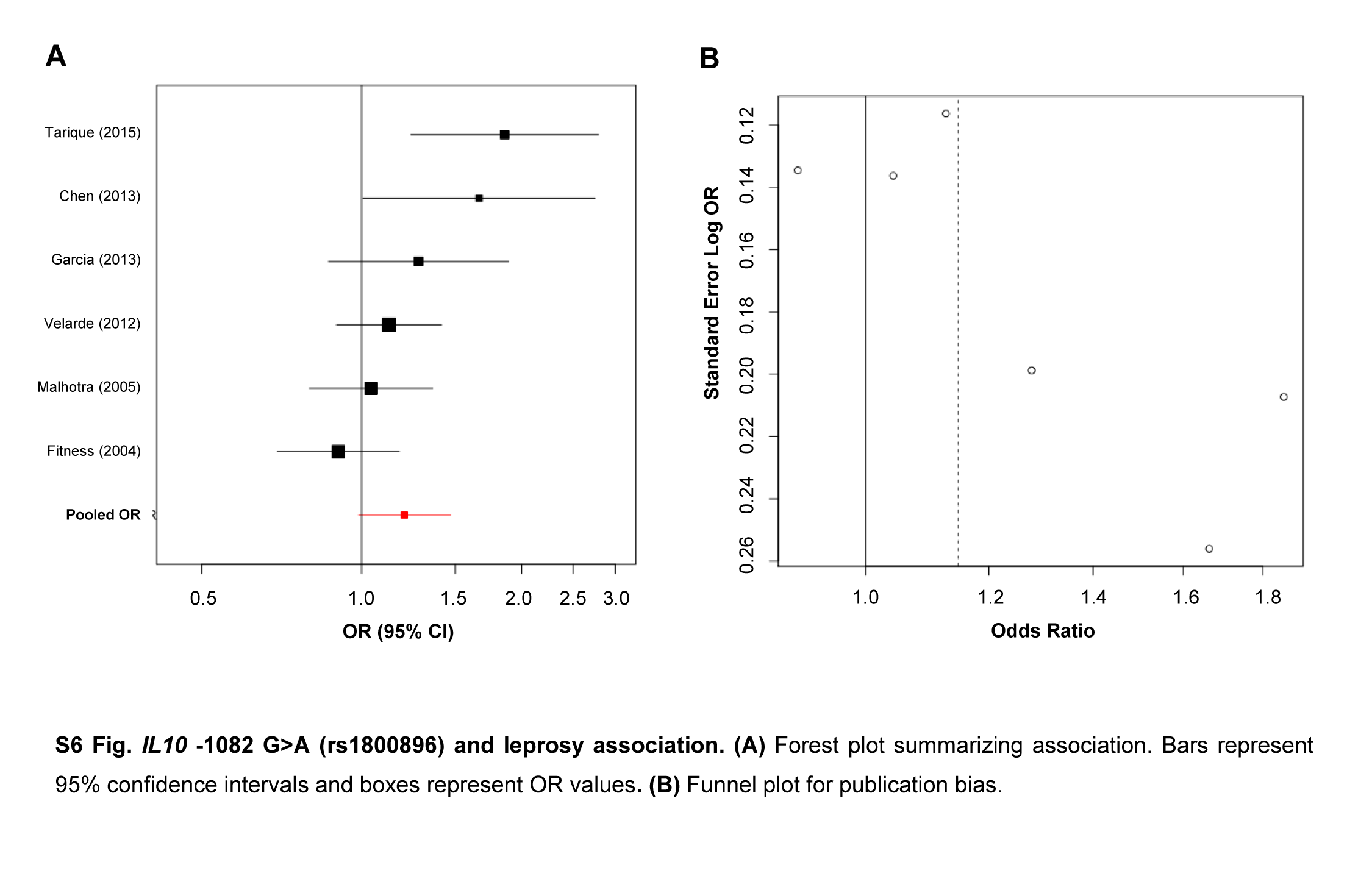

Supplement: S6 Fig — (A) Forest plot summarizing association. Bars represent 95% confidence intervals and boxes represent OR values. (B) Funnel plot for publication bias (TIF) [file pone.0136282.s007.tif]

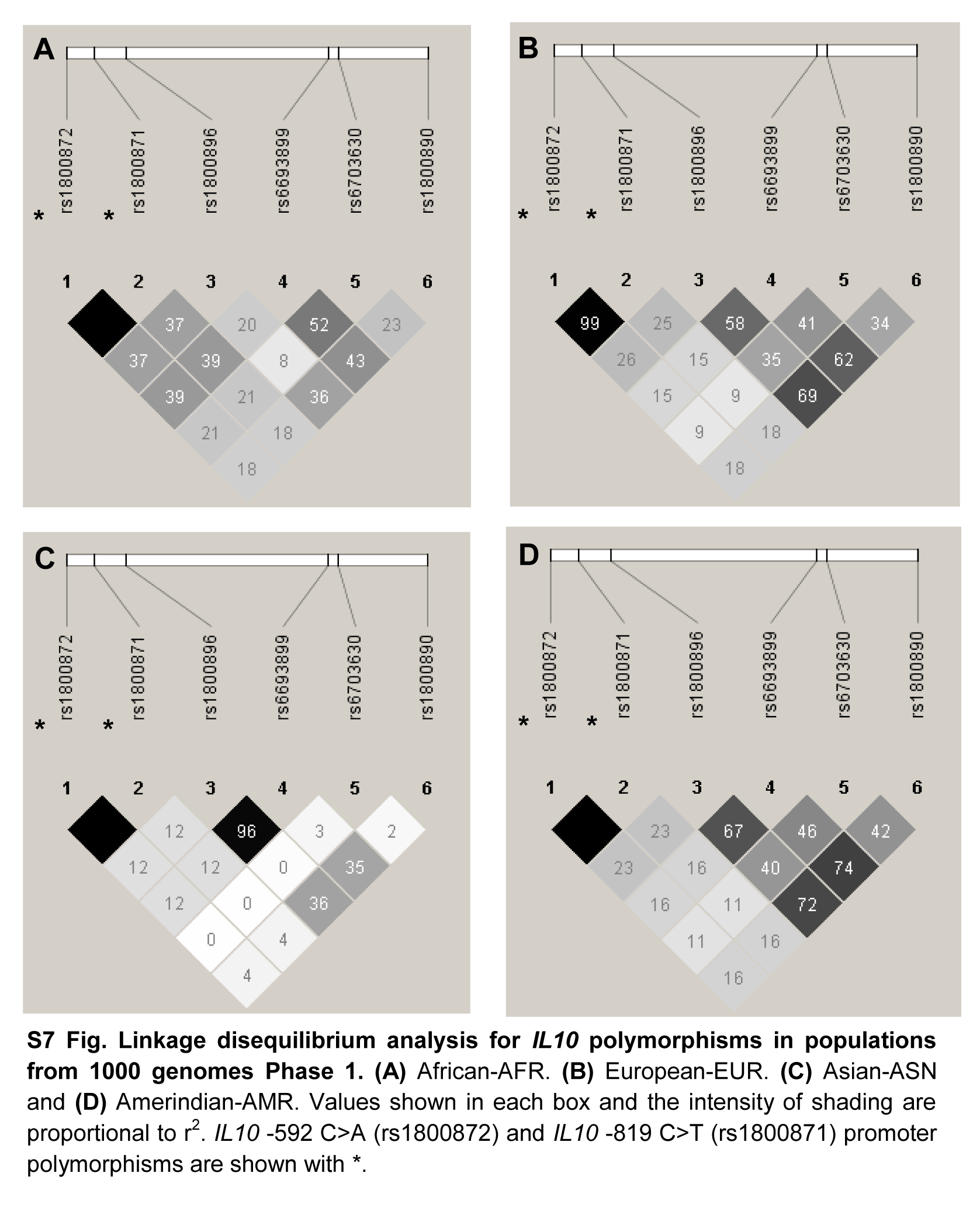

Supplement: S7 Fig — (A) African-AFR. (B) European-EUR. (C) Asian-ASN and (D) Amerindian-AMR. Values shown in each box and the intensity of shading are proportional to r2. IL10 –592 C>A (rs1800872) and IL10 –819 C>T (rs1800871) promoter polymorphisms are shown with *. (TIF) [file pone.0136282.s008.tif]
