# Supplementary material for: Association of IL10 Polymorphisms and Leprosy: A Meta-Analysis
Source: PLoS One. 2015 Sep 4;10(9):e0136282. doi: 10.1371/journal.pone.0136282 (PMC4560376; doi:10.1371/journal.pone.0136282)
Supplement: S2 Table — (DOC) [file pone.0136282.s010.doc]

**S2 Table. Characteristics of the studies included in the meta-analysis for the *IL10* polymorphisms and leprosy**.

| **Study** | | **Cases** | | | | | **Controls** | | | | | **Genotyping method** |
| --- | --- | --- | --- | --- | --- | --- | --- | --- | --- | --- | --- | --- |
| **First author, year** | **Population** | **Age^a^** | **N** | **Females** | **Males** | **MB/PB^b^** | **Age^a^** | **N** | **Females** | **Males** | **Source^c^** |
| **Santos et al., 2002** | Brazil | 49.3±7.3 | 300 | 113 | 187 | 210/90 | 47.4-4.1 | 92 | 60 | 32 | BB | RFLP |
| **Moraes et al., 2004** | Brazil | - | 301 | 104 | 197 | 166/131 | - | 295 | 185 | 110 | HI | RFLP |
| **Fitness et al., 2004** | Malawi | - | 270 | - | - | 26/244 | - | 452 | - | - | HI | RFLP |
| **Malhotra et al., 2005** | India | 30.4±3.2 | 282 | 48 | 238 | 140/142 | 28.2±4.1 | 266 | 44 | 122 | HI | Sequencing |
| **Pereira et al., 2009** | Brazil | 41±18 | 380 | - | - | 291/79 | 36±11 | 369 | - | - | BB | RFLP |
| **Aggarwal et al., 2011** | India | 32.3 | 807 | 182 | 552 | 379/355 | 36 | 1640 | 442 | 852 | HI | MS |
| **Velarde et al., 2012** | México | 24-88 | 68 | 21 | 47 | * | 18-62 | 144 | 75 | 69 | BB | RFLP |
| **Cardona et al., 2012** | Colombia | 42.5 | 100 | 36 | 64 | 25/75 | 41.3 | 100 | 72 | 28 | HC | SSP |
| **Garcia et al., 2013** | Brazil | 43.4 ± 1.8 | 138 | - | - | 108/30 | 23.0 ± 0.5 | 96 | - | - | HI | TaqMan |
| **Chen et al., 2013** | China | 46 ± 18 | 193 | - | - | 131/62 | 40 ± 18 | 189 | - | - | HI | TaqMan and Sequencing |
| **Tarique et al., 2015** | India | 41.3 ± 12.8 | 102 | 31 | 71 | ND | 35.8 ± 10.6 | 120 | 37 | 83 | HI | SSP |

Abbreviations: N, total counts; MB, Multibacilary; PB, Paucibacilary; SD, Standard deviation; -, Data not available

^a^ Age is described either as mean ± SD, or range when – is present

^b^ Number of MB patients / Number of PB patients

^c^ Source of Control Group individuals: BB= Blood bank donors, HC= Unrelated household contacts, HI = Healthy individuals living in the same geographical area but unrelated to case group

ND = No description

*All patients in Velarde et al., 2012 were MB
